# Lithium’s effects on therapeutic targets and MRI biomarkers in Parkinson’s disease: A pilot clinical trial^☆^

**DOI:** 10.1016/j.ibneur.2023.05.001

**Published:** 2023-05-07

**Authors:** Thomas Guttuso, Rachel Shepherd, Luciana Frick, M. Laura Feltri, Valerie Frerichs, Murali Ramanathan, Robert Zivadinov, Niels Bergsland

**Affiliations:** a Department of Neurology, Clinical and Translational Science Institute, Jacobs School of Medicine and Biomedical Sciences, University at Buffalo, Buffalo, NY, United States; b Department of Chemistry, Clinical and Translational Science Institute, Jacobs School of Medicine and Biomedical Sciences, University at Buffalo, Buffalo, NY, United States; c Department of Pharmaceutical Sciences, Clinical and Translational Science Institute, Jacobs School of Medicine and Biomedical Sciences, University at Buffalo, Buffalo, NY, United States; d Center for Biomedical Imaging, Clinical and Translational Science Institute, Jacobs School of Medicine and Biomedical Sciences, University at Buffalo, Buffalo, NY, United States; e IRCCS, Fondazione Don Carlo Gnocchi, Milan, Italy

**Keywords:** Lithium, Biomarker, Free water, Cognition, Progression, Clinical trial

## Abstract

**Background:**

Lithium has a wide range of neuroprotective actions, has been effective in Parkinson’s disease (PD) animal models and may account for the decreased risk of PD in smokers.

**Methods:**

This open-label pilot clinical trial randomized 16 PD patients to “high-dose” (*n* = 5, lithium carbonate titrated to achieve serum level of 0.4–0.5 mmol/L), “medium-dose” (*n* = 6, 45 mg/day lithium aspartate) or “low-dose” (*n* = 5, 15 mg/day lithium aspartate) lithium therapy for 24-weeks. Peripheral blood mononuclear cell (PBMC) mRNA expression of nuclear receptor-related-1 (Nurr1) and superoxide dismutase-1 (SOD1) were assessed by qPCR in addition to other PD therapeutic targets. Two patients from each group received multi-shell diffusion MRI scans to assess for free water (FW) changes in the dorsomedial nucleus of the thalamus and nucleus basalis of Meynert, which reflect cognitive decline in PD, and the posterior substantia nigra, which reflects motor decline in PD.

**Results:**

Two of the six patients receiving medium-dose lithium therapy withdrew due to side effects. Medium-dose lithium therapy was associated with the greatest numerical increases in PBMC Nurr1 and SOD1 expression (679% and 127%, respectively). Also, medium-dose lithium therapy was the only dosage associated with mean numerical decreases in brain FW in all three regions of interest, which is the opposite of the known longitudinal FW changes in PD.

**Conclusion:**

Medium-dose lithium aspartate therapy was associated with engagement of blood-based therapeutic targets and improvements in MRI disease-progression biomarkers but was poorly tolerated in 33% of patients. Further PD clinical research is merited examining lithium’s tolerability, effects on biomarkers and potential disease-modifying effects.

## 1 Introduction

Parkinson’s disease (PD) is the second most common and fastest growing neurodegenerative disorder with a worldwide prevalence that is predicted to more than double over the next 25 years (Dorsey and Bloem, 2018). Although there are several FDA-approved dopaminergic therapies to mask the motor symptoms of PD, no therapy has been shown to slow the progressive worsening of motor symptoms nor the onset of dementia that occurs, on average, within 10 years of diagnosis and represents the most disabling long-term PD sequela (Aarsland et al., 2000; Schrag et al., 2000; Hely et al., 2008; Lawson et al., 2016). In order to identify therapies that can slow disease progression (both motor and cognitive symptom progression) and improve PD patients’ prognosis, a.k.a “disease-modifying therapies”, such therapies will first need to show positive effects on therapeutic targets and known disease-progression biomarkers.

Lithium has multiple neuroprotective actions including suppressing microglial activation, reducing inflammation and oxidative stress, and enhancing autophagy and mitochondrial biogenesis and function (Sarkar et al., 2005; Struewing et al., 2007; Arraf et al., 2012; Dong et al., 2014; Hou et al., 2015). Lithium treatment has demonstrated benefit in several PD animal models (Youdim and Arraf, 2004; Kim et al., 2011; Lieu et al., 2014; Zhao et al., 2019). Also, the 77% risk reduction of PD in smokers shown in prospective cohort studies has been theorized to be due to the high levels of lithium in tobacco (Guttuso et al., 2019). Based on this background, we performed a pilot clinical trial in PD to assess lithium’s ability to engage blood-based therapeutic targets and MRI-based disease-progression biomarkers in order to help determine if further clinical research on lithium’s potential disease-modifying effects in PD was merited.

A PD therapeutic target of interest for over 25 years is the nuclear receptor-related 1 protein (Nurr1) (Zetterstrom et al., 1996, Lin et al., 2012, Jeon et al., 2020), which is a transcriptional cofactor that upregulates the expression of genes essential for dopamine neuron differentiation and survival (Le et al., 1999; Decressac et al., 2013). Nurr1 immunoreactive substantia nigra (SN) neurons decrease by 46% with aging (Chu et al., 2002), which is the main risk factor for PD. In PD patients, Nurr1 immunoreactive SN neurons are reduced by 65% and Nurr1 mRNA by 61% in peripheral blood mononuclear cells (PBMCs) compared to aged-matched healthy controls (Chu et al., 2006; Li et al., 2018). Nurr1 is inversely correlated with intraneuronal alpha-synuclein and ameliorates alpha-synuclein-mediated dopamine cell toxicity (Yang and Latchman, 2008; Decressac et al., 2012; Lin et al., 2012; Li et al., 2018). Nurr1 also decreases the expression of alpha-synuclein and reduces inflammation stemming from microglia (Yang and Latchman, 2008; Saijo et al., 2009). One of the genes under the control of Nurr1 is superoxide dismutase-1 (SOD1) (Volakakis et al., 2010), which is a primary antioxidant brain enzyme. In PC12 cells, lithium treatment increases Nurr1 by about 180% and protects against rotenone-induced death (Zhang et al., 2016). Additional blood-based PD therapeutic targets that lithium may engage include serum brain-derived neurotrophic factor (BDNF), plasma alpha-synuclein and the inhibited form of glycogen synthase kinases-3β phosphorylated at serine 9 (pS9-GSK3β), in PBMCs (Li et al., 2007; Leyhe et al., 2009; Lin et al., 2017).

Because it is unknown if lithium’s ability to engage these blood-based therapeutic targets confers any disease-modifying effects in PD, we also explored if lithium therapy was associated with improvements in known MRI-based disease-progression biomarkers. Previous analyses of a University of Florida (UF) and the Parkinson’s Progression Markers Initiative (PPMI) longitudinal PD biomarker cohorts have shown a diffusion-based MRI assessment called free water (FW) in the posterior substantia nigra (pSN) to longitudinally reflect progressive worsening of motor symptoms (Ofori et al., 2015; Burciu et al., 2017). A more recent PPMI analysis showed increasing FW in the dorsomedial nucleus of the thalamus (DMN-T) and nucleus basalis of Meynert (nbM) to reflect progressive worsening of cognition in PD (Guttuso et al., 2022). FW corresponds to water molecules within a voxel that are not hindered or restricted by the cellular environment and therefore originate from extracellular water (Pasternak et al., 2009). FW is believed to reflect tissue atrophy, cell death and inflammation (Pasternak et al., 2012, Burciu et al., 2017, Febo et al., 2020), which are known to occur in PD. Thus, therapies shown to slow FW progression in these sites would represent promising disease-modifying therapies for slowing motor and cognitive decline in PD.

Considering that lithium dosages as low as 0.3 mg/day may have disease-modifying effects in neurodegenerative disease and high dosages are poorly tolerated in PD (Coffey et al., 1984, Nunes et al., 2013, Guttuso et al., 2019), we studied three relatively low lithium dosages compared to those required for treating bipolar disorder, lithium’s only FDA-approved indication. For bipolar disorder, an elemental lithium dosage of about 300 mg/day is needed to produce a therapeutic serum level of 0.8–1.0 mmol/L. “Elemental lithium” refers to the weight of lithium alone not including its salt carrier.

## 2 Materials and methods

The University at Buffalo’s Institutional Review Board approved the study prior to patient enrollment (Clinicaltrials.gov: NCT04273932). Eligible patients were 45–80 years old diagnosed with PD by the UK Brain Bank Diagnostic Criteria under the care of the principal investigator, did not have dementia or formed visual hallucinations, no history of stroke or brain surgery, had stable PD medications for > 30 days and psychiatric medications for > 60 days with no current need for adjustments, stable or no diuretic or NSAID use for > 30 days, no history of prescription or dietary supplement lithium use, no history of nilotinib or a glucagon-like peptide-1 agonist use, no use of tobacco for > 1 year, normal thyroid stimulating hormone (TSH) level and estimated glomerular filtration rate ≥ 50. After providing written informed consent, 16 patients were randomized using a random number table to either “high-dose” (*n* = 5, lithium carbonate titrated to achieve a trough serum level of 0.4–0.5 mmol/L), “medium-dose” (*n* = 6, 45 mg/day elemental lithium aspartate) or “low-dose” (*n* = 5, 15 mg/day elemental lithium aspartate) lithium therapy for 24 weeks. Three additional PD patients who chose not to receive lithium therapy served as controls. Fasting blood samples were obtained at baseline, 12 and 24 weeks. For the high-dose group, dose titration was based on trough serum lithium levels assessed by Kaleida Health Laboratories, Williamsville, NY. For all patient groups, trough serum lithium levels were assessed by inductively coupled plasma mass spectrometry (ICPMS) by the UB Chemistry Instrument Center using 6-lithium as an internal standard, which provided a limit of quantification of 1.0 µg/L.

As this was a pilot study with limited funding, a step-wise approach was used to prioritize the blood-based therapeutic target assessments. In the initial eight patients enrolled, PBMC Nurr1 and SOD1 mRNA, serum BDNF and plasma alpha-synuclein were assessed to determine the most promising blood-based therapeutic targets to assess in the remaining patients. PBMC Nurr1 and SOD1 mRNA were assessed by Taqman quantitative, real-time polymerase chain reaction (ThermoFisher Scientific, Waltham, MA). Values were normalized to actin levels. Serum BDNF was assessed by ELISA kit (R&D Systems, Minneapolis, MN). Plasma alpha-synuclein was assessed by ultra-sensitive, immunomagnetic reduction assay (MagQu, Taiwan). PBMC GSK3β (total and serine 9 phosphorylated (pS9) levels) were assessed by PathScan ELISA kit (Cell Signaling Technology, Danvers, MA) in the patients who had adequate PBMC protein to support these assays. In the final nine patients, only PBMC Nurr1 and SOD1 were assessed as these showed the most promise in the initial eight patients. With this approach, enough funding was preserved to obtain MRI scans at baseline and 24 weeks in six patients receiving lithium (two from each lithium dosage group chosen consecutively).

All MRI scans were obtained on a Canon Vantage Titan 3 T scanner using a 32-channel head coil. The MRI protocol included an MPRAGE acquisition (1 mm^3^ isotropic voxels) along with a multi-shell diffusion weighted imaging acquisition (11 b = 0 s/mm^2^ volumes, 21 directions at b = 1000 s/mm^2^ and 25 directions at b = 2000 s/mm^2^ with 2.7 mm^3^ isotropic voxels) and 11,819 ms repetition time. A b= 0 image was acquired with opposite phase encoding. Diffusion-weighted images were corrected for susceptibility-induced and eddy current/subject movement-induced distortions using a combination of the FSL tools topup (Andersson et al., 2003) and eddy (Andersson and Sotiropoulos, 2016) Next, the corrected diffusion-weighted data were processed with the free water elimination model (Hoy et al., 2014) implemented in DIPY (https://dipy.org) to obtain FW maps.

3D T1-weighted images were corrected for intensity inhomogeneity using the N4 tool and subsequently segmented using the SIENAX tool (Smith et al., 2001) to obtain partial volume estimate maps of the gray matter. The nbM was then segmented as previously described (Schulz et al., 2018). Briefly, a histologically defined map of the nbM (Eickhoff et al., 2005) was brought into the native 3D T1-weighted image space for each scan via non-linearly warping with Advanced Normalization Tools (ANTs) (Klein et al., 2009). In addition, each 3D T1-weighted image was processed using the FreeSurfer 6.0 pipeline (Fischl et al., 2004) along with the thalamic nuclei segmentation submodule (Iglesias et al., 2018). Outputs were visually inspected for errors and misclassification. Manual corrections to the FreeSurfer output (e.g., introduction of white matter control points, editing of brain mask and/or white matter mask) were made as appropriate. For obtaining a mask of the DMN-T region of interest (ROI), the following individual nuclei were combined into a single segmentation: paratenial, reuniens (medial ventral), mediodorsal medial magnocellular and mediodorsal lateral parvocellular. The pSN ROI was obtained using previously published methodology (Yang et al., 2019). Briefly, the mean b= 0 image was non-linearly registered with ANTs to MNI space, where a standard space ROI of the pSN was defined. The resulting ROIs were then brought back into native space using the corresponding inverse transformation. Finally, the nBM and DMN-T maps were brought into the diffusion space using boundary based registration (Greve and Fischl, 2009) between the 3D T1-weighted image and the b= 0. The maps of the nBM, DMN-T, and pSN in diffusion space were used to obtain free water values within each ROI. Reported FW values are the mean of the bilateral sites for each ROI.

For comparison, we reprocessed the diffusion data using the MarkVCID script, while limiting the input to the b= 1000 shell and correlated the FW values with those derived from the multi-shell acquisition data. We also re-analyzed the pSN FW data using manual ROI placement drawn in the native space of each diffusion acquisition, without referencing the MNI-defined pSN ROIs, and calculated the correlation coefficient.

## 3 Results

Two of the six patients randomized to medium-dose lithium therapy withdrew from the study both due to side effects of sedation, incoordination and slowness of thinking. None of the other patients reported any side effects. (After the study was completed, one of the medium-dose patients who withdrew from the study resumed lithium aspartate at 30 mg/day and reported no side effects at a steady-state trough serum lithium level of <0.1 mmol/L but again experienced side effects when the dosage was increased to 45 mg/day).

Patient demographics for the 17 patients who completed the study are reported in Table 1.

**Table 1:** Patient demographics for study completers.

Lithium Dose	Age (years)	Sex (M/F)	Disease Duration (years)	Levodopa Equivalent Dose (Tomlinson et al., 2010)	MoCA	MDS-UPDRS-III (“on”)
**High** (*n* = 5)	63.8	4/1	2.76	615	27.4	21.6
**Medium** (*n* = 4)	62	1/3	2.75	325	26.3	16.0
**Low** (*n* = 5)	66.6	3/2	5.95	870	27.0	21.8
**Control PD** (*n* = 3)	69.7	3/0	6.33	1283	25.7	22.7

All values are means. PD: Parkinson’s disease, MoCA: Montreal Cognitive Assessment, MDS-UPDRS-III (“on”): Movement Disorder Society-Unified Parkinson’s Disease Rating Scale-Part III assessed in the “on” state.

Mean changes in blood-based therapeutic targets are reported in Table 2. Mean trough serum lithium levels at 24 weeks were 1282, 828, 195 and 0.73 µg/L in the high-dose, medium-dose, low-dose and control PD patients, respectively.

**Table 2:** Mean 24-week % changes in blood-based therapeutic targets.

Lithium Dose	PBMC Nurr1 Expression	PBMC SOD1 Expression	Serum alpha-Synuclein	Serum BDNF	PBMC pS9/total GSK3β
**High-Dose**	420% (n = 5)	18% (n = 5)	-5% (n = 2)	-15% (n = 2)	421% (n = 2)
**Medium-Dose**	679% (n = 4)	127% (n = 4)	34% (n = 2)	-25% (n = 2)	17% (n = 3)
**Low-Dose**	93% (n = 5)	-1% (n = 5)	-3% (n = 2)	-29% (n = 2)	260% (n = 1)
**Control PD**	139% (n = 3)	8% (n = 3)	-12% (n = 2)	-28% (n = 2)	93% (n = 1)

PD: Parkinson’s disease, PBMC: peripheral blood mononuclear cell, Nurr1: nuclear receptor-related 1 protein, SOD1: superoxide dismutase 1, BDNF: brain-derived neurotrophic factor, GSK3β: glycogen synthase kinase-3 beta.

Because increasing Nurr-1 mRNA expression by about 180% was shown to protect against rotenone-induced death in PC12 cells (Zhang et al., 2016), patients having a > 200% increase in Nurr1 mRNA expression at 24 weeks were defined as “Nurr1 responders”. With this definition, the Nurr1 responder rates for the high-, medium-, and low-dose lithium groups and controls were 20%, 75%, 20% and 0%, respectively. SOD1 mRNA expression at 24 weeks changed by 125% and −21% in Nurr1 responders and non-responders, respectively.

Mean disease duration for the low-dose, medium-dose and high-dose lithium-treated patients who received MRIs were 1.3 years, 1.5 years and 3.5 years, respectively. Correlations between the multi-shell FW values and those obtained using the MarkVCID script with only the b= 1000 shell data exceeded 0.943 for all of the ROIs. There was an intra-class correlation coefficient of 0.94 between pSN FW data using MNI-defined and manual pSN ROI placement.

Mean changes in FW in the six PD patients who received baseline and 24-week MRIs are reported in Table 3 in addition to 1-year changes in FW in PD patients and age-matched healthy controls from the UF and PPMI longitudinal cohorts for comparison.

**Table 3:** Mean FW longitudinal changes from pilot lithium/PD trial, UF and PPMI longitudinal cohorts.

	Mean Change in DMN-T FW	Mean Change in nbM FW	Mean Change in pSN FW
**High-Dose Lithium** (24-Week Changes)	-0.0117 (*n* = 2)	-0.0140 (*n* = 2)	0.0198 (*n* = 2)
**Medium-Dose Lithium** (24-Week Changes)	-0.0098 (*n* = 2)	-0.0416 (*n* = 2)	-0.0307 (*n* = 2)
**Low-Dose Lithium** (24-Week Changes)	0.0123 (*n* = 2)	-0.0306 (*n* = 2)	0.0787 (*n* = 2)
**PD from UF** (1-Year Changes)	---	---	0.038 (Ofori et al., 2015) (*n* = 25)
**Aged-Matched HCs from UF** (1-Year Changes)	---	---	0.001 (Ofori et al., 2015) (*n* = 19)
**PD from PPMI** (1-Year Changes)	0.0100 ± 0.030 (Guttuso et al., 2022) (*n* = 130)	0.0025 ± 0.041 (Guttuso et al., 2022) (*n* = 130)	0.0180 ± 0.055 (Burciu et al., 2017) (*n* = 103)
**Aged-Matched HCs from PPMI** (1-Year Changes)	0.0005 ± 0.024 (Guttuso et al., 2022) (*n* = 58)	-0.0085 ± 0.034 (Guttuso et al., 2022) (*n* = 58)	0.0070 ± 0.030 (Burciu et al., 2017) (*n* = 49)

FW: free water, PD: Parkinson’s disease, UF: University of Florida, PPMI: Parkinson’s Progression Markers Initiative, DMN-T: dorsomedial nucleus of the thalamus, nbM: nucleus basalis of Meynert, pSN: posterior substantia nigra, HC: healthy control.

**Fig. 1:**
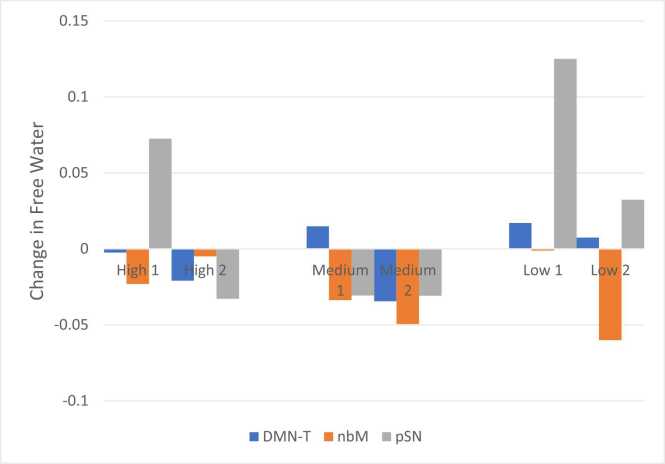
Individual patient changes in FW after 24 weeks of high, medium or low-dose lithium therapy. FW: free water, DMN-T: dorsomedial nucleus of the thalamus, nbM: nucleus basalis of Meynert, pSN: posterior substantia nigra.

The percent of PD patients treated with medium-dose lithium aspartate for 24 weeks showing longitudinal FW decreases of at least 0.02 in the DMN-T, nbM and pSN were 50%, 100% and 100%, respectively (Fig. 1), compared to 13%, 22% and 38% of PD patients from PPMI, respectively, over one year.

## 4 Discussion

Out of the three lithium dosages tested in this small pilot trial, medium-dose lithium aspartate therapy was associated with the largest numerical increases in expression of blood-based PD therapeutic targets Nurr1 and SOD1 and the most uniform improvements in the MRI-based disease-progression biomarker FW in sites previously shown to reflect progressive motor and cognitive decline in PD (Ofori et al., 2015; Burciu et al., 2017; Guttuso et al., 2022). However, two of the six patients receiving this lithium dosage withdrew from the study due to side effects while none of the high- or low-dose lithium patients reported any side effects. Because lithium’s side effects are typically dose-dependent, it is unclear what accounted for these findings considering that the mean serum lithium levels were 55% higher in the high-dose group compared to the medium-dose group.

We hypothesize that lithium may dissociate from the salt carrier aspartate more readily than from carbonate and/or cross cellular barriers more readily resulting in higher serum and/or intraneuronal “free lithium” levels at equivalent elemental lithium dosages of the two formulations. Because commercial lithium assays typically assess total, not free lithium levels, biological assays like PBMC Nurr1 expression represent a more objective way to compare dose-response relationships among different lithium formulations. This study’s findings of numerically higher magnitudes of PBMC Nurr1 and SOD1 mRNA expression associated with medium-dose lithium aspartate versus high-dose lithium carbonate support this hypothesis. In addition, one of the medium-dose lithium aspartate patients who withdrew due to side effects when receiving 45 mg/day subsequently reported no side effects when receiving 30 mg/day with a steady-state trough serum lithium level < 0.1 mmol/L. At 45 mg/day of lithium aspartate, the highest predicted serum lithium level in this patient would be about 0.15 mmol/L, which would be extremely unlikely to produce side effects if derived from lithium carbonate therapy. In support, a recent study showed 10–100-fold disparate potencies of several different lithium formulations when assessed on an array of biological assays (Torshin et al., 2022). Patient characteristics including levodopa equivalent dose and disease duration did not appear to contribute to the two patients withdrawals. Further research is needed examining the effects of different lithium formulations including lithium aspartate on lithium-sensitive biological assays. In order to minimize patient withdrawals, future clinical research on lithium aspartate therapy could utilize a dose titration schedule up to a maximum tolerated dosage of 30–45 mg/day based on an individual’s side effects.

Because several studies have shown pSN FW to be a disease-progression biomarker in PD (Ofori et al., 2015, Burciu et al., 2017), the monoamine oxidase B (MAO-B) inhibitor rasagiline, which has several neuroprotective actions, was recently studied in a randomized controlled trial in PD to assess its effects on this outcome (Arpin et al., 2021). The results showed rasagiline to not significantly affect longitudinal pSN FW progression; however, the placebo group did have over a 4-fold higher 1-year increase in pSN FW compared to the rasagiline group. Our findings of longitudinal reductions in pSN FW associated with medium-dose lithium aspartate therapy for 24 weeks contrasts the findings from this study with rasagiline. Although rasagiline has several neuroprotective mechanisms of action, it has never been shown to affect Nurr1 expression. Lithium, on the other hand, does not inhibit MAO-A or MAO-B and robustly increases Nurr1 expression in PC12 cells (Nag, 2004; Zhang et al., 2016). Lithium also has anti-inflammatory and autophagy-enhancing actions that have not been described for rasagiline.

Although motor symptom progression leads to disability in PD, cognitive impairment with progression to dementia has a greater negative impact on quality of life in PD than motor symptoms, occurs in the majority of PD patients and strongly predicts nursing home placement (Aarsland et al., 2000; Schrag et al., 2000; Hely et al., 2008; Lawson et al., 2016). On average, it takes about 10 years for dementia to occur after PD diagnosis (Aarsland and Kurz, 2010), which highlights the need for identification of cognition progression biomarkers that could be used in clinical trials to help identify therapies to slow cognitive decline even in early PD. Recently, our group identified FW progression in the DMN-T and nbM to reflect longitudinal cognitive decline in early PD from PPMI (Guttuso et al., 2022). Thus, a therapy that slowed FW progression in the DMN-T and nbM in PD would imply that it may be able to slow long-term cognitive decline and prevent dementia. Our findings associating medium-dose lithium aspartate therapy with longitudinal reductions in DMN-T and nbM FW show promise towards these ends.

The biggest shortcoming of our pilot study was its very small sample size, which may have led to spurious findings and prevented any meaningful intergroup statistical comparisons. In addition to the small sample size, only a subset of enrolled patients was able to receive MRI scans due to funding limitations. Although baseline pSN FW and patient disease duration did not appear to influence the FW results, it is difficult to make any firm conclusions based on such a small *n.* Therefore, these data should be considered preliminary until a larger number of patients can be studied. Future studies could also explore if lithium’s effects on biomarkers varies in patients carrying genetic variants known to cause PD or increase PD risk.

## 5 Conclusions

Medium-dose lithium aspartate therapy was associated with strong engagement of blood-based therapeutic targets and improvements in MRI disease-progression biomarkers in PD but also with a high patient withdrawal rate due to side effects. Further clinical research is merited on lithium’s tolerability, effects on these biomarkers and potential disease-modifying effects in PD.

## Ethical approval

Compliance with Ethical Standards: The authors declare that all experiments on human subjects were conducted in accordance with the Declaration of Helsinki https://www.wma.net/policies-post/wma-declaration-of-helsinki-ethical-principles-for-medical-research-involving-human-subjects/ and that all procedures were carried out with the adequate understanding and written consent of the subjects. The authors also certify that formal approval to conduct the experiments described has been obtained from the human subjects review board at the University at Buffalo and can be provided upon request.

## Funding

A Buffalo Blue Sky Award from the 10.13039/100008209University at Buffalo, a private donation and NSF CHE-0959565. The funding sources had no role in study design; in the collection, analysis or interpretation of data; in the writing of the report; or in the decision to submit the article for publication.

## CRediT authorship contribution statement

**Thomas Guttuso Jr.:** Research project, Conception, Organization, Execution, Manuscript Preparation, Writing of the first draft, Review and Critique. **Rachel Shepherd:** Research project, Organization, Execution, Manuscript Preparation, Review and Critique. **Luciana Frick:** Research project, Organization, Execution, Manuscript Preparation, Review and Critique. **Laura Feltri:** Research project, Execution, Manuscript Preparation, Review and Critique. **Valerie Frerichs:** Research project, Execution, Manuscript Preparation, Review and Critique. **Murali Ramanathan:** Research project, Execution, Manuscript Preparation, Review and Critique. **Robert Zivadinov:** Research project, Execution, Manuscript Preparation, Review and Critique. **Niels Bergsland:** Research project, Organization, Execution, Manuscript Preparation, Writing of the first draft, Review and Critique.

## Conflicts of interest

Authors’ Conflicts of Interest for previous 12 months: Thomas Guttuso, Jr.: President of e3 Pharmaceuticals, Inc. Support from UCB for clinical trial patient enrollment. Rachel Shepherd: None. Luciana Frick: None. Laura Feltri: None. Valerie Frerichs: None. Murali Ramanathan: None. Robert Zivadinov: Received personal compensation from Bristol Myers Squibb, EMD Serono, Sanofi, Novartis, Sanofi, 415 Capital, Mapi Pharma and Janssen for speaking and consultant fees. He received financial support for research activities from Bristol Myers Squibb, Sanofi, Novartis, EMDSerono, V-WAVE Medical, Mapi Pharma, CorEvitas and Protembis. Niels Bergsland: None.
